# Malnutrition and gender disparities in the Eastern Mediterranean Region: The need for action

**DOI:** 10.3389/fnut.2023.1113662

**Published:** 2023-03-07

**Authors:** Jana Jabbour, Merette Khalil, Anna Rita Ronzoni, Ruth Mabry, Ayoub Al-Jawaldeh, Maha El-Adawy, Hala Sakr

**Affiliations:** ^1^Nutrition Program, Department of Natural Sciences, Lebanese American University, Beirut, Lebanon; ^2^Department of Healthier Populations, Regional Office for the Eastern Mediterranean, World Health Organization, Cairo, Egypt; ^3^Global Public Health Consultant, Muscat, Oman; ^4^Department of Mental Health and Non-Communicable Diseases, Regional Office for the Eastern Mediterranean, World Health Organization, Cairo, Egypt

**Keywords:** malnutrition, inequity, gender disparities, Eastern Mediterranean Region, obesity, wasting (malnutrition)

## Abstract

Malnutrition takes a heavy toll on the populations of the Eastern Mediterranean Region (EMR), with gender related socioeconomic risk factors impacting undernutrition and obesity in both women and men. This perspective article, a derivative of a report by the World Health Organization, reviews the scientific literature on the effect of gender on malnutrition related outcomes in the EMR. Results revealed that biological and gender-related socioeconomic risk factors play a role for undernutrition and obesity in both women and men. Malnutrition can be negatively influenced by gender-biased cultural standards, habits, structural determinants, differential exposures, and health system gaps. This can result, for example, in women tending to focus on familial and household related needs, at the expense of their own health and physical mobility and on suffering more food insecurity, undernutrition, micronutrient deficiencies and obesity compared to men in the EMR. Conflict and crisis situations negatively affect both genders, but generally put women at a higher risk of adverse. Women’s socially limited autonomy in mobility is also an obstacle to access to health services in the EMR, including those related to nutrition. Multi-level approaches are needed to address gender issues to enable a more equitable distribution of resources and reduce the impact of malnutrition in the EMR.

## Background

The double burden of malnutrition, the co-existence of under nutrition and obesity, affects nearly 2.3 billion people globally (1). Malnutrition takes a heavy toll on the health and well-being of the population of the Eastern Mediterranean Region (EMR), a heterogenous Region with wide economic, social, cultural and health disparities among countries (2). While some EMR countries, especially those affected by conflict, continue to experience high levels of food insecurity, undernutrition, and micronutrient deficiencies, others have been experiencing a nutrition transition (Table 1), with a shift toward unhealthy diets and sedentary lifestyles resulting in 53% of women, 45% of men and 8% of school-age children or adolescents in the Region being obese, respectively (5).

**Table 1:** Classification of countries in the Eastern Mediterranean Region according to the stage of nutrition transition and the World bank income classification (3, 4).

World bank income classification/Stage of nutrition transition	High-income	Middle-income (including upper and lower middle-income countries)	Low-income
Advanced	Bahrain, Kuwait, Oman, Qatar, Saudi Arabia, UAE	Iran	
Early		Egypt, Jordan, Lebanon, Morocco, Palestine, Tunisia	
Triple burden of malnutrition		Iraq, Pakistan	Djibouti
Complex emergencies		Libya, Syria	Afghanistan, Somalia, Sudan, Yemen

Malnutrition has social, economic and environmental determinants and the stark health inequalities between and within countries explain the wide variation in the prevalence of under and over-nutrition in the EMR (6, 7). Gender is a key determinant of health, resulting from a combination of both sex-linked biology and gender-related social influences on health outcomes including malnutrition (8, 9). Gender inequality adversely affects the health of women and men, girls and boys as it influences exposure to gender-biased cultural standards, norms and expectations, structural causes, differential exposures and vulnerabilities and health system gaps (10). In relevance to malnutrition, gender inequality has been linked with a greater percentage of low birth weight infants and childhood mortality around the world (11). Childhood stunting has recently shown to have a heavy health cost on productivity in North Africa and the Middle east, costing 1,035–1,339 million dollars of monthly loss for the private sector (12).

**Figure 1:**
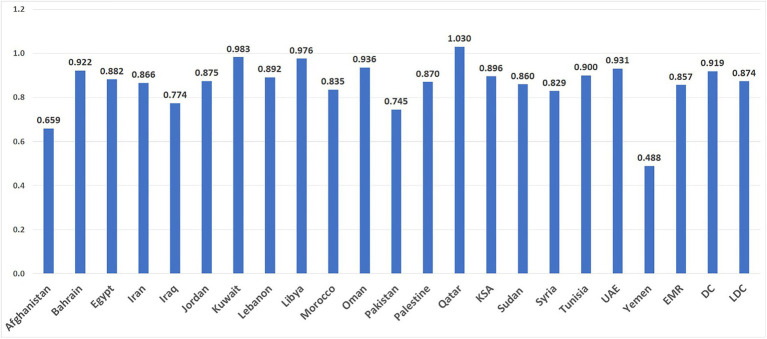
The Gender Development Index (GDI) is an indicator of the gender gap assessing the male and female ratios for the following indicators: Life expectancy at birth, expected years of schooling, mean years of schooling, and estimated gross national income per capita. Higher GDI scores reflect greater human and gender development (13) DC: Developing Countries; EMR: Average of countries in the Eastern Mediterranean Region; LDC: Least Developed Countries. KSA: Kingdom of Saudi Arabia; UAE: United Arab Emirates.

Recent gender analyzes by the United Nations Development Program showed that the EMR’s Gender Development Index (GDI), a measure of the gender gap in human development is below the world average and that of the least developed countries (13). Within the EMR, the GDI was highest in Qatar and lowest in Yemen (5) (Figure 1). The life expectancy of women in the EMR is 71 years compared to 68 years in men. Yet, women are expected to live 1.7 years of these 3 years in illness (5); this gender paradox reflects how even though women live longer than men, they face higher rates of illness (14). Health inequalities are partly driven by and intersect with gender differences in educational attainment, labor force participation as well as other determinants of health like class, age and disability status (15).

This paper explores the underlying risk factors and consequences of gender discrimination and the unequal power dynamics contributing to malnutrition in the Region and identifies public health actions and literature gaps for further exploration at the intersection of gender and malnutrition. This article is a derivative of a larger project and report on gender and health conducted by the WHO Regional Office of the EMR which included available evidence between years 2000 and 2019 (16).

### Conceptual framework

The analysis of the interplay between gender and malnutrition in the WHO regional report and in this manuscript is guided by Sen and Ostlin’s conceptual framework, which describes both structural and intermediary determinants of gendered health outcomes (15, 16). Structural determinants can be in the form of economic, social, and/or political forces. Intermediary factors comprise the gender norms and power dynamics stemming from discriminatory standards, practices, and behaviors as well as differential exposure and vulnerabilities to diseases, which are connected to biases in health systems and in research. Power structures impact different aspects of people’s lives including health and the complexity of such impact can only be understood through the intersectional conceptualization of gender as a social determinant of health (15).

### Prevalence of malnutrition in the EMR

EMR countries are in different phases of the nutrition transition depending on their socio-economic, political and urbanization contexts as well as the extent of technological development, access to information, food processing and mass media growth (17). Given the heterogeneity between countries, an elucidation of where countries stand in the nutrition transition process helps the reader better understand the link between gender and the different forms of malnutrition (Table 1). The Gulf Cooperation Council countries are in the advanced phase characterized by an elevated prevalence of obesity, a moderate prevalence of undernutrition and selected micronutrient deficiencies (18). Countries such as Egypt, Jordan, Lebanon, Morocco, Palestine, and Tunisia are classified in an ‘early’ nutrition phase with a moderate prevalence of obesity (even though increasing) and undernutrition (4). Countries such as Djibouti, Iraq and Pakistan have a triple burden of malnutrition with the coexistence of acute and chronic malnutrition, micronutrient deficiencies and an increase in obesity rates (18). Countries such as Afghanistan, Libya, Somalia, Sudan, Syria and Yemen are going through complex emergency situations characterized by severe child and maternal malnutrition and prevalent micronutrient deficiencies (4).

**Figure 2:**
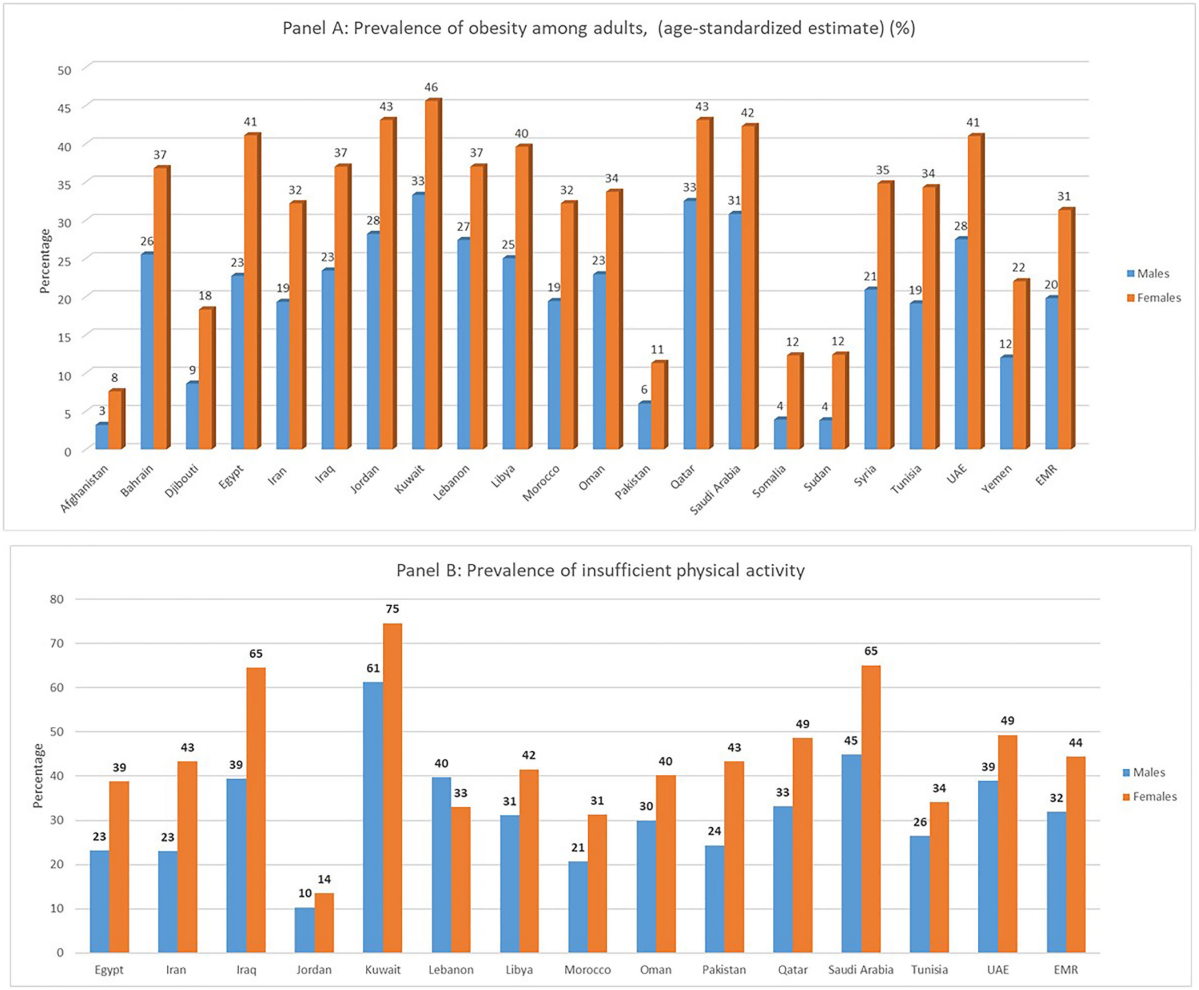
Prevalence of age-standardized obesity Panel **(A)** and insufficient physical activity Panel **(B)** among adults aged 18 years or older, by country and sex in the Eastern Mediterranean Region (EMR) (24).

The overall regional prevalence of undernutrition is estimated to be 19%. The highest levels of stunting, wasting, and underweight (among children) are observed in Afghanistan, Djibouti, Pakistan, Somalia, Sudan, and Yemen (19, 20). Micronutrient deficiencies due to low intake and/or status are common in the EMR. It has been estimated that for every three EMR residents, one has micronutrient deficiencies (4). EMR countries record a high rate of iron deficiency and anemia ranging from 20 to 47% among women of reproductive age (15–49 years) (5, 19). Even though most of its countries are considered sunny, the EMR bears a heavy burden of Vitamin D deficiency (21), with up to 83% of EMR residents being Vitamin D deficient, as compared to less than 20% of North Europe residents where it is less sunny (22, 23). While EMR countries, especially those affected by conflict, continue to experience high levels of food insecurity, undernutrition, and micronutrient deficiencies, physical inactivity, overweight and obesity are also common (Figure 2). In the EMR, 47% of adults are overweight or obese compared to 39% globally. The differences between men and women is wider compared to the worldwide rates (53% in women vs. 45% in males in the EMR compared to 40% vs. 39% in women and men, respectively, in the world) (25).

### Social cultural, structural and behavioral determinants of malnutrition

Gender is a key determinant of malnutrition resulting from a combination of both sex-linked biology and gender-related social influences on health outcomes (8). Across the region, women’s triple burden of productive, reproductive, and social roles is associated with more time dedicated to familial and household nutrition-related needs and less for attending to their own needs. At the same time, gender norms limit women’s physical mobility and further exacerbate gender and health inequalities in malnutrition (16).

In its socio-economic and political heterogeneity, the EMR is characterized by conflict-related emergency and humanitarian contexts, which play a major structural role in malnutrition. For instance, in Yemen, where the ongoing conflict has resulted in the displacement of around 3.3 million people, malnutrition and undernutrition currently affect 3 million people including 1.5 million children, 1.1 million pregnant and lactating women, and 400,000 acutely malnourished children, putting them at high risk of famine (19). Gender inequality in malnutrition can be further exacerbated when combined with other factors such as conflict or crisis, generally putting women at higher risk of more adverse outcomes. For example, collecting water or food can expose women to gender-based violence, leaving them and their children at increased risk of undernutrition and malnutrition, as a consequence of the inability to perform those tasks (26). In humanitarian crises, women and girls are more vulnerable to malnutrition as the social norms can result in them eating less during food scarcity in favor of men and boys (26). Women, in fact, tend to reduce their own food intake in order to allow other family members to eat, especially male children and men (26, 27). On the other hand, while men and boys are favored when it comes to access to food, they can also suffer from malnutrition during conflict or crisis owing to a lack of cooking skills and access to food distribution (28).

### Risk factors

Women and girls have increased biological, socioeconomic, and behavioral risk factors for malnutrition than men and boys. Firstly, with regards to the biological risk factors, women require more protein, energy and micronutrients than usual when pregnant and lactating (29). In view of women’s cyclical loss of iron during menstruation and childbearing time, their nutrition status is particularly vulnerable to micronutrient deficiencies (29). EMR countries record a high rate of iron deficiency and anemia, which ranges from 20 to 47% among women of reproductive age (15–49 years) (5). The highest rates were reported among Egyptian women (47%), adolescents (47%), and children (38%), and the lowest were found among Saudi school-aged children (12%) and elderly (13%) (30). In terms of biological risk factors for obesity, men have a higher likelihood of storing visceral fat compared to pre-menopausal women, which increases their risk of developing type 2 diabetes, cardiovascular events, and death (31, 32). Females have higher deposits of subcutaneous fat than men and see their stores of adipose tissues and visceral fat increase after menopause, heightening the adverse events associated with obesity (33).

Secondly, in relation to socioeconomic factors, some of the household-level predictors of stunting among women in the Region are their low status, inadequate dietary habits during pregnancy and lactation, inappropriate intra-household food allocation, widespread poverty, and poor access to clean water (34, 35). A study from Pakistan found correlations between malnutrition and low-income households and identified lack of maternal literacy to be strongly correlated with malnutrition (36). In relation to vitamin D, being a woman has been identified as a significant predictor of Vitamin D deficiency in the Region with rates of deficiencies reaching 96% in some countries (37, 38). This is due to social and religious variables like skin covering and veiling that constitute a risk factor for Vitamin D deficiency in most, though not all, studies in the EMR (23, 37, 39–44). Women of lower socio-economic status have increased risk of malnutrition due to their limited access to healthy foods, stressful lifestyles, and limited physical activity (25, 45). Notably, the socioeconomic factors driving malnutrition in the EMR reflect a complex reality as obesity rates are highest in some of the Region’s wealthiest countries: Kuwait (42.5% in men, 47.1% in women), Qatar (39.5% in men, 43.2% in women), and Bahrain (32.3% in men, 40.3% in women) (46).

Finally, behavioral risk factors do affect malnutrition differently. In the EMR, one of the top contributing risk factors to obesity and NCDs is the high prevalence of physical inactivity (47). It is estimated that the EMR is the second highest region in the prevalence of physical inactivity globally (35%), with women being less active than men in most countries (48). One study found that physical inactivity ranges from 60% in Jordan, to 95% in Egypt and 98% in KSA (49). Males have been found to engage in more physical activity than women in most EMR countries except for Lebanon (50). In Jordan, one study revealed higher inactivity rates among males while the WHO data reflected the opposite (24, 51). Further to this, when disaggregating for sex, young women in the region have higher rates of physical inactivity compared to the global figures (52). The social limitations of women’s physical mobility in the Region constitute a major factor for inactivity (53). For example, youth centers in Egypt catering for lower socio-economic groups were criticized for being open to male youth only, although on paper they were supposed to be accessible to both sexes (54).

### Access to health care services

Women’s socially limited autonomy in mobility not only exacerbate ‘lifestyle’ risk factors such as food consumption and physical inactivity, but also represent one of biggest obstacles to access and utilization of health services in the Region. These gender-related social norms not only limit women’s mobility but also restrict autonomy in decision-making regarding their health and well-being. One study from Afghanistan found that the lack of autonomy for women is an important obstacle to antenatal visits (55). The most recent regional health profile indicated that antenatal care is insufficient in the EMR, with coverage of at least four clinic visits for antenatal care is under 80% for 10 countries, and as low as 3.3% in Somalia (5).

Another barrier toward gender-sensitive universal access to health is the lack of culturally acceptable health services, including the scarcity of female health providers to attend female patients. For example, a study in Jordan, found that some women favored private service providers rather than public, in part due to the increased availability of female obstetricians; however, those attending private providers used public hospitals for birth, which creates discontinuity and fragmentation in maternal-neonatal healthcare, and in child healthcare services (56).

### Health outcomes

Health outcomes from malnutrition can be serious and wide-ranging (25). Across the EMR, iron deficiency is one of the leading determinants of adolescent disability-adjusted life years, especially in low- and middle-income countries, contributing to 20% of maternal deaths (25). Moreover, maternal malnutrition has been linked to low birth weight, and growth retardation in children (57, 58), which in turn results in high infant morbidity and mortality rates, adding to health care costs and undermining the economic human resource potential nationally (59). Malnutrition in mothers jeopardizes the quality of care they can offer their children by reducing the meaningful mother–child interaction that is necessary for proper growth (60). Experience from low-income and conflict-affected countries in the EMR like Yemen, Somalia, Afghanistan, Sudan and Djibouti shows that poor female nutrition early in life reduces learning potential, increases reproductive and maternal health risks, and lowers productivity (2, 7). This situation contributes to women’s diminished ability to gain access to other assets later in life and undermines attempts to eliminate gender inequalities (61). In essence, women with poor nutrition are caught in a vicious circle of poverty and malnutrition.

The effects of obesity and overweight on health outcomes are also complex and multifaceted. Overweight and obesity increase the risk in both men and women of chronic diseases such as hypertension, diabetes and is associated with a higher mortality (62, 63). Although obesity has negative health outcomes among both men and women, studies have shown that obese women are more likely to develop diabetes than obese men (64). However, obese men are more likely to develop chronic pulmonary disease and chronic kidney disease than women. Obesity also has a bidirectional relationship with mental illnesses. Individuals with a mental illness have an increased risk of obesity; and obesity is associated with psychiatric disorders including but not limited to mood disorders, anxiety, and personality disorders (65). The association of obesity and depression can be particularly significant among women (66).

## Policy implications

This perspective piece summarized the malnutrition chapter of the WHO’s regional report on gender and health in the EMR (16). This paper is among the first of its kind to utilize a gender-analysis of nutrition disparities across the Region. Given the timeframe of the original report, it is unfortunate that some articles may have been missed for this piece. The limited availability of age and sex-disaggregated data, along with limited gender-analyzes, is another limitation affecting the understanding of the root-causes behind the epidemiological trends and overall health disparities across the Region (5, 10, 16). Nevertheless, on the basis of the solid findings featured in this manuscript, policymakers and practitioners alike would benefit from the below recommendations.

Given the co-existence of under and over-nutrition in countries of the Region, and even within households, policies and nutrition interventions should target specific age and gender groups (16). At the same time, efforts are needed to improve the overall quality of household diets ensuring that all members have access to healthy foods. Improving water and sanitation infrastructure and supporting women to exclusively breast-feed babies would go a long way in not only promoting health and well-being but also reducing underweight and stunting among children and addressing the intergenerational cycle of malnutrition (16). However, a much stronger region-specific evidence base is critical in identifying the most appropriate combination of interventions and policies that would successfully address gender and malnutrition in the EMR. As reflected in the regional gender and health report, routine gender analysis and in-depth understanding of gender differences in dietary habits and their determinants can guide the development of gender-sensitive nutrition policies and culturally acceptable health services. A gendered approach to food security during conflict is also necessary to address malnutrition issues (16).

Because of their triple productive, reproductive, and social roles, poor female nutrition can have substantial effects on our community. Malnutrition among women negatively affects women’s learning potential and productivity and increases reproductive, maternal and children’s health risks. It is hence of primary importance to identify means of eliminating relevant gender inequalities. (9, 26). Addressing gender discrimination and power inequalities can improve women’s status including her own nutritional status and that of their children (16). Recommendations to address gender inequalities include working toward a paradigm shift to eradicate the structural obstacles underlying the problem in the region (16). A comprehensive and multilevel approach to confront the power dynamics underpinning gender inequities in the various socio-cultural systems is much needed (67).

## Conclusion

Malnutrition in the EMR is a leading health issue resulting in various health inequalities. This review described the interaction between gender and malnutrition and identified the serious impact of gendered systems and norms on malnutrition in the EMR. Gender norms and stereotypes as well as gender-based inequalities have been associated with a higher prevalence of food insecurity, undernutrition, micronutrient deficiencies and obesity, and have contributed to worse health outcomes, with differing manifestations for both men and women. The gender dimension in malnutrition intersects with individual determinants such as lack of education, unemployment, and low income as well as structural determinants such as culture, socio-economic factors, and political stability. It is therefore of primary importance to address malnutrition from a gender perspective. An intersectoral, multi-level, gender-sensitive approach is needed to reduce the impact of malnutrition and address gender-related health inequalities in the EMR.

## Data availability statement

The original contributions presented in the study are included in the article/supplementary material, further inquiries can be directed to the corresponding author.

## Author contributions

AA-J, ME-A, JJ, MK, RM, AR, and HS: conceptualization. JJ, MK, RM, AR, and HS: methodology. JJ, MK, RM, AR, and HS: investigation. JJ, MK, RM, AR, and HS: writing–original draft preparation and writing–review and editing. All authors have read and agreed to the published version of the manuscript.

## Conflict of interest

The authors declare that the research was conducted in the absence of any commercial or financial relationships that could be construed as a potential conflict of interest.

## Publisher’s note

All claims expressed in this article are solely those of the authors and do not necessarily represent those of their affiliated organizations, or those of the publisher, the editors and the reviewers. Any product that may be evaluated in this article, or claim that may be made by its manufacturer, is not guaranteed or endorsed by the publisher.
